# In vitro biosynthesis of ATP from adenosine and polyphosphate

**DOI:** 10.1186/s40643-021-00469-0

**Published:** 2021-11-29

**Authors:** Chuanqi Sun, Zonglin Li, Xiao Ning, Wentian Xu, Zhimin Li

**Affiliations:** 1 State Key Laboratory of Bioreactor Engineering, East China University of Science and Technology, 130 Meilong Road, Shanghai, 200237 China; 2 Shanghai Collaborative Innovation Center for Biomanufacturing Technology, 130 Meilong Road, Shanghai, 200237 China

**Keywords:** Adenosine triphosphate, In vitro, Multi-enzymatic cascade catalysis, Polyphosphate kinase, Adenosine kinase

## Abstract

Adenosine triphosphate (ATP) acts as a crucial energy currency in vivo*,* and it is a widely used energy and/or phosphate donor for enzyme-catalyzed reactions in vitro. In this study, we established an in vitro multi-enzyme cascade system for ATP production. Using adenosine and inorganic polyphosphate (polyP) as key substrates, we combined adenosine kinase and two functionally distinct polyphosphate kinases (PPKs) in a one-pot reaction to achieve chain-like ATP regeneration and production. Several sources of PPK were screened and characterized, and two suitable PPKs were selected to achieve high rates of ATP production. Among these, *Sulfurovum lithotrophicum* PPK (SlPPK) exhibited excellent activity over a wide pH range (pH 4.0–9.0) and synthesized ATP from ADP using short-chain polyP. Furthermore, it had a half-life > 155.6 h at 45 °C. After optimizing the reaction conditions, we finally carried out the coupling-catalyzed reaction with different initial adenosine concentrations of 10, 20, and 30 mM. The highest yields of ATP were 76.0, 70.5, and 61.3%, respectively.

**Graphical Abstract:**

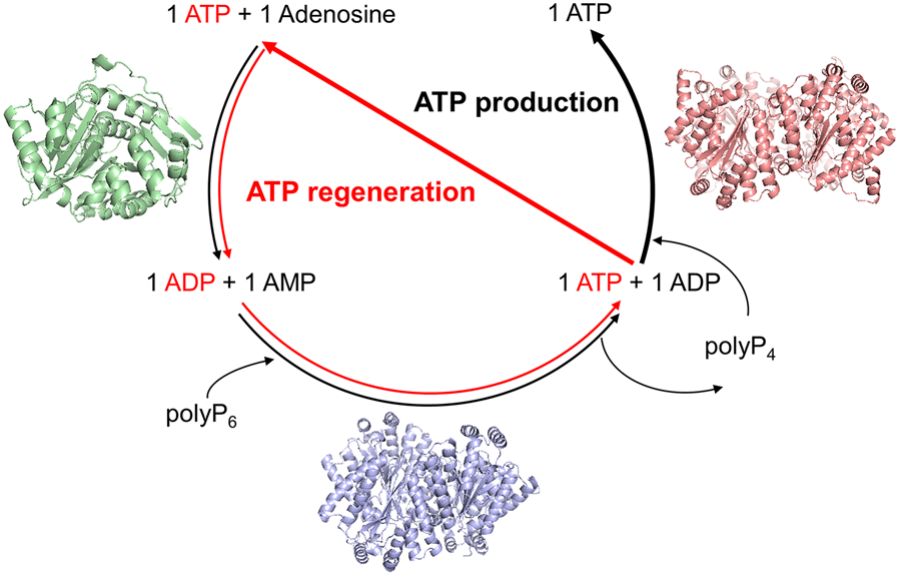

**Supplementary Information:**

The online version contains supplementary material available at 10.1186/s40643-021-00469-0.

## Introduction

Adenosine triphosphate (ATP) is an energy currency containing high-energy phosphate bonds. It plays a central role in numerous aspects of the cellular metabolism (Nath 2016; Chi Da and Kido 2014), such as in the biosynthesis of proteins, lipids, and nucleotides, and providing energy for active transport mechanisms (Chen and Zhang 2021). It is also required in numerous in vitro enzyme reactions as the energy provider (Kitao and Hata 2018; Huang et al. 2019; Praetorius et al. 2010; Sperl et al. 2018). ATP plays a key role in signal transduction (Rajendran et al. 2016), as it is involved in the synthesis of intracellular second messenger cyclic adenosine monophosphate (cAMP) (Post et al. 1998). Furthermore, extracellular ATP has a good effect on the treatment or adjuvant treatment of cancer, heart disease, hepatitis, and other diseases (Vultaggio et al. 2020; Dou et al. 2018; Stagg and Smyth 2010).

Several methods of ATP production have been developed. In the twentieth century, ATP was extracted from metabolically active rabbit and insect muscles (Calaby 1951; Baddiley et al. 1948). The extracted ATP could be formulated into injection for clinical use. However, this method was too costly for scalable production. Later, chemical synthesis of ATP was established using phosphorylation reagents and chemical catalysts; however, this causes serious environmental pollution, and the structure and optical properties of the final product were difficult to guarantee (Koichiro et al. 2007). Currently, ATP is synthesized using living yeast cells on an industrial scale (Kreil and Hoffmann 1963; Kadowaki et al. 1989; Asada et al 1978). In this process, glucose is added to provide energy for the ATP production process through the glycolytic pathway. Nevertheless, the energy utilization of this method is low, and the yeast enzyme system with different batches exhibits large differences in productivity (Yao et al. 2011). ATP has been used as medicine and for industrial biosynthesis of costly medicine precursors, but its market price of about 150 US dollars per kilogram remains too costly. In recent years, many of the high value-added products such as cosmetics, pharmaceuticals, and some important intermediates are produced through enzymatic biosynthesis, which significantly reduces production costs. Compared with the chemical and microbial fermentation method, enzymatic biosynthesis has numerous advantages, such as fast speed, high yield, easy control, and optimization (Becker et al. 2021; Cheng et al. 2019; Bai et al. 2019). Nevertheless, reports on the enzymatic synthesis of ATP are lacking.

Polyphosphate kinase (PPK, EC 2.7.4.1) is widely used in the ATP regeneration system, as its substrate inorganic polyphosphate (polyP) has the advantages of high stability and low cost (Shimane et al. 2012; Meng et al. 2016; Li et al. 2020a, b). Two PPK families have been widely characterized (Tavanti et al. 2021). The enzymes belonging to the PPK 1 family are generally used to catalyze the extension reaction of polyP chains, and their abilities to regenerate ATP are relatively poor (Nocek et al 2018). In contrast, PPK 2 enzymes tend to catalyze the synthesis of polyP-driven nucleoside phosphate rather than degradation, and they show no sequence similarity and are structurally unrelated to the PPK 1 family (Mordhorst and Andexer 2020; Batten et al. 2015; Parnell et al. 2018). The PPK 2 family is further divided into three classes according to the phylogenetic analysis and substrate preference of enzymes: Class I catalyzes the phosphorylation of adenosine diphosphates (ADP) to ATP; Class II prefers to use adenosine monophosphates (AMP) as a substrate to produce ADP, and Class III provides catalytic activity for the above two reactions (Ogawa et al. 2019). Some PPK2-III enzymes catalyze the synthesis of adenosine polyphosphates, such as adenosine tetraphosphate (A4P) and adenosine pentaphosphate (A5P) (Mordhorst et al. 2019). Some PPKs, such as *Sinorhizobium meliloti* PPK, *Francisella tularensis* PPK (belonging to PPK 2-I), *Acinetobacter johnsonii* PPK (belonging to PPK 2-II), and *Meiothermus ruber* PPK (belonging to PPK 2-III) have also been found to have the properties of adenylate kinase (Ogawa et al. 2019).

In this study, an in vitro multi-enzyme cascade system was constructed that simultaneously performs the regeneration and synthesis of ATP, which results in the net accumulation of ATP. The corresponding adenosine kinase (Adk, EC 2.7.1.20) and PPK 2 belonging to Class III catalyzed ATP regeneration, while PPK 2 belonging to Class I was used for ATP synthesis. After screening the suitable PPKs, the temperature, pH, magnesium ion concentration, and enzyme concentration of the coupling reaction were optimized to achieve in vitro production of ATP at different adenosine concentrations.

## Materials/experimental

### Strains, plasmids, chemicals, and culture media

*Escherichia coli* TOP10 was used for plasmid amplification and preservation. *E. coli* Rosetta (DE3) and the plasmid pET28a ( +) were used for protein expression. All chemicals were purchased from Macklin and Aladdin (Shanghai, China). The Luria–Bertani (LB) medium supplemented with 50 μg/mL kanamycin was used for *E. coli* cell growth and recombinant protein expression.

### Construction of the expression system

The *atadk* gene of *Arabidopsis thaliana* (NP_195950.1) and the six *ppk2* genes of *Sulfurovum lithotrophicum* (WP_046551064.1), *Desulfurella amilsii* (WP_086034570.1), *Acidithiobacillus caldus* (WP_004871423.1), *Lampropedia hyalina DSM 16,112* (SHF67157.1), *Rhizobacter sp. OV335* (SHM26193.1), and *Acidovorax sp. OV235* (PYG87866.1) were synthesized by Sangon Biotech (Shanghai, China) and introduced into the *Bam HI/Xho I* restriction sites of pET28a ( +), yielding the plasmids pET28a-*atadk*, pET28a-*slppk*, pET28a-*dappk*, pET28a-*acsppk*, pET28a-*lhppk*, pET28a-*rsppk*, and pET28a-*acppk*. To enhance the gene expression level, DNA sequences were codon-optimized for *E. coli* (Additional file 1: Table S1). Then, the recombinant plasmids were transformed into *E. coli* Rosetta (DE3) for enzyme production.

### Expression and purification of enzymes

Expression and purification of the enzymes (AtAdk, SlPPK, DaPPK, AcsPPK, LhPPK, RsPPK, AcPPK) were performed as described previously (Shen et al. 2019). The recombinant cells were incubated in 3 mL of LB medium at 37 °C and 220 rpm for 6–8 h. Next, 2 mL of the cultures was transferred to 100 mL of newly prepared LB medium in 500-mL flasks and cultivated at 37 °C. Overexpression of the enzymes was induced by the addition of 0.2 mM isopropyl-β-d-thiogalactopyranoside when the optical density at 600 nm of the culture reached 0.6–0.8, and then, the bacteria were incubated at 18 °C and 220 rpm for 16 h. After induction, the cells were harvested by centrifugation at 4600 × g and 4 °C for 5 min, washed once and resuspended in 10 mM Tris–HCl buffer (pH 8.0). The cells were then lysed by a high-pressure homogenizer supplied by Shanghai Litu Mechanical Equipment Engineering Co., Ltd. (Shanghai, China). The lysate was centrifuged at 6000 × g and 4 °C for 20 min. The protein in the supernatant was purified by Ni-chelation affinity chromatography. The purity of the enzyme was examined by SDS-PAGE, and the proteins were quantified by the BCA Protein Assay Kit from Tiangen (Beijing, China).

### Enzyme activity assays

The activity of PPKs at 37 °C was assayed in 100 mM Tris–HCl buffer (pH 8.0) containing 10 mM AMP or ADP, 20 mM MgCl_2_, 20 mM hexametaphosphate (polyP_6_), and an appropriate amount of enzymes. The enzymes of PPK2-I used ADP as substrate and the PPKs of class III used both AMP and ADP as substrate. Routine assays were performed with 5 min of incubation and immediately terminated by adding an equal volume of 1 M HCl on ice. The samples were centrifuged at 13,500 × g and 4 °C for 10 min, then the supernatant were taken to dilute and detected by high-performance liquid chromatography (HPLC, see HPLC analysis method). One unit of PPKs activity was defined as the amount of enzyme that released 1 μmol of product per minute.

### Effect of phosphate donor concentration

The effect of the polyP_6_ concentration on the specific activity of AtAdk was tested. The reactions were conducted in 100 mM Tris–HCl buffer (pH 8.0) at 45 °C containing 10 mM Adenosine, 10 mM ATP, 20 mM MgCl_2_, appropriate amount of enzymes and different concentrations of polyP_6_ that ranged from 10 to 50 mM. The effect of the polyP_6_ concentration on the specific activity of LhPPK was also tested. The reactions were conducted in 100 mM Tris–HCl buffer (pH 8.0) at 45 °C containing 10 mM ADP, 20 mM MgCl_2_, appropriate amount of enzymes and different concentrations of polyP_6_ that ranged from 10 to 50 mM. Both the two reactions were stopped after 5 min by adding an equal volume of 1 M HCl on ice. The samples were, respectively, diluted and analyzed by measuring the concentration of ADP or ATP using HPLC. The inhibitory effect of polyP_6_ on AtAdk or LhPPK activity was tested. The enzyme activity at the lowest polyP_6_ concentration was set to 100% of the relative activity. The utilization of PPKs to polyP_6_ was tested. The reaction was carried out in 100 mM Tris–HCl buffer (pH 8.0) at 45 °C containing 10 mM ADP, 1 mM polyP_6_, 20 mM MgCl_2_ and appropriate amount of enzymes. The reaction was stopped after 2 h by adding an equal volume of 1 M HCl on ice. Samples were diluted and analyzed by HPLC.

### Optimization of temperature and pH

The effect of pH on the first stage reaction was determined at 45 °C in buffers with different pH values ranging from 5.0 to 9.0: phosphate buffer (PB) buffer (pH 5.0 − 7.0), and Tris–HCl buffer (pH 7.0 − 9.0). The highest activity of the enzyme under different pH conditions was set as 100% of relative activity. The optimal temperature on the first stage reaction was determined at different temperatures from 37 to 50 °C. Adenosine was consumed in 100 mM Tris–HCl buffer (pH 8.0) at 37, 45, and 50 °C, respectively. Samples were taken at different point times to observe the overall situation of the reaction.

### Thermostability

The thermostability of SlPPK was tested by incubating in 100 mM Tris–HCl buffer at 45 and 50 °C. The same amount of enzyme was taken at different times to determine the residual enzyme activity. The initial enzyme activity was defined as 100%. The first-order kinetic reaction model was used to estimate the inactivation rate constant of enzyme inactivation (Eq. 1):

1 $$\ln A = \, \ln A_{0} - kt$$

where *A* is the relative enzyme activity after thermal incubating for *t* h, *A*_*0*_ is the initial relative enzyme activity, and *k* is the inactivation rate constant (h^−1^).

### Optimization of the enzyme amount

The enzyme loading amounts of AtAdk and LhPPK were optimized at 45 °C by changing their ratios from 1:4 to 12:1 and simultaneously keeping the total amount of enzymes unchanged. The 200 μl reaction system contained 10 mM Adenosine, 20 mM polyP_6_, 2 mM ATP, 20 mM MgCl_2_ and 12 mg enzymes in 100 mM Tris–HCl buffer (pH 8.0). Samples was taken after 10 min and used to measure the rate of adenosine consumption to determine the optimum enzyme ratio.

### Multienzyme coupling reaction

The reaction system for ATP production contained 20 mM polyP_6_, 0.5 mM ATP, 20 mM MgCl_2_ and AtAdk and LhPPK in 100 mM Tris–HCl buffer (pH 8.0) at different initial adenosine concentration from 10 to 30 mM. The total amount of the two enzymes added was 0.1 g/L, and the ratio of AtAdk: LhPPK was 4:1. 0.1 g/L SlPPK was added at the moment when the adenosine was completely consumed. The concentration changes of ATP, ADP, AMP, and adenosine were detected by HPLC in the first process.

### HPLC analysis

The concentrations of ATP, ADP, and AMP was determined through HPLC with a C18 column (4.6 × 150 mm, Wondasil, Shimadzu-GL). The mobile phase was composed of 50 mM monopotassium phosphate: 100% methanol (97:3, V/V) at a flow rate of 0.6 mL/min. The column temperature was maintained at 30 °C, and the detection wavelength was set at 270 nm. The composition of the mobile phase for adenosine detection was 50 mM monopotassium phosphate: 100% methanol (90:10, v/v) at a flow rate of 0.8 mL/min. Samples were first diluted in water. They were then filtered (0.22-μm hydrophilic PTFE syringe filter, ANPEL, Shanghai). Finally, 10 µL was applied to the column. The data were analysed with Dionex Chromeleon software (Additional file 1: Figure S2).

## Results and discussion

### Overview of designed pathway

**Fig. 1:**
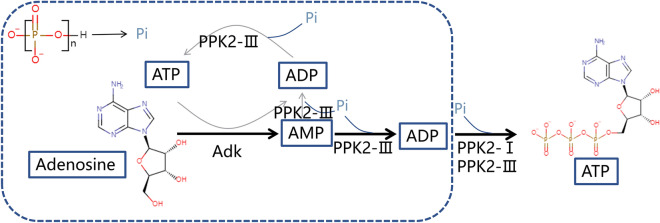
Designed pathway of the in vitro synthetic enzymatic biosystem for the production of ATP. The Pi for providing phosphate group is from polyP_6_. Three enzymes are used in system. Among them, adenosine kinase catalyzes the reaction of adenosine and ATP to generate AMP and ADP, PPK2-I catalyzes ADP and polyP_n_ to generate ATP and polyP_(n-1)_, PPK2-III catalyzes AMP to generate ADP and then from ADP to generate ATP

To synthesize ATP from adenosine, a multi-enzyme cascade catalytic system containing AtAdk and PPKs was constructed in vitro (Fig. 1). The production of ATP was accomplished through gradual phosphorylation of adenosine. The first step was the conversion of adenosine to AMP by AtAdk, which required ATP as a phosphate donor. Therefore, a small amount of ATP was necessary to initiate the coupling reaction and conversion to ADP. AMP was then phosphorylated into ADP and ATP by PPK using polyP_6_ as a phosphate donor. ATP generated in the early stage would continue to participate in the phosphorylation of adenosine until the adenosine in the system was completely consumed. Then, ATP began to accumulate through the action of PPK. To facilitate the subsequent description, the adenosine consumption stage is defined as the first stage, and the second stage is the ATP production process. AtAdk from *Arabidopsis thaliana* in the literature was selected to phosphorylate adenosine, and the optimal reaction conditions for this enzyme are pH 8.0–9.5 and 37 °C (Moffatt et al. 2000). The conversion of adenosine reached 100% by AtAdk, and no adenosine could be detected in the HPLC results (data not shown).

### Screening and characterization of PPKs

To find suitable PPKs for coupled reactions, we screened enzymes from Class I and III of the PPK2 family that were often used in ATP regeneration. According to the previously study in our laboratory, PPK derived from Acidibacillus sulfuroxidans (AsPPK) possesses good thermostability but low activity (Li et al. 2020a, b). Using the sequence of AsPPK as a template, Basic Local Alignment Search Tool (BLAST) was used to search for candidate sequences. Three Class I PPKs were randomly selected from the results, namely, DaPPK, AcsPPK, and SlPPK. For the screening of class III enzymes, LhPPK, RsPPK, and AcPPK were selected directly from the NCBI database (Table 1). The codon-optimized PPKs were synthesized and overexpressed in *E. coli* Rosetta (DE3) in LB medium. SDS-PAGE results showed that all PPKs were expressed solubly and purified (Additional file 1: Figure S1).

**Table 1:** PPK2 enzymes screened in this work

GenBank	Short name	Organism	Transformation	Class
SHF67157.1	LhPPK	*Lampropedia hyalina DSM 16112*	AMP → ATP	2-III
PYG87866.1	AcPPK	*Acidovorax sp. OV235*	AMP → ATP	2-III
SHM26193.1	RsPPK	* Rhizobacter sp. OV335*	AMP → ATP	2-III
WP_046551064.1	SlPPK	*Sulfurovum lithotrophicum*	ADP → ATP	2-I
WP_004871423.1	AcsPPK	*Acidithiobacillus caldus*	ADP → ATP	2-I
WP_086034570.1	DaPPK	*Desulfurella amilsii*	ADP → ATP	2-I

**Fig. 2:**
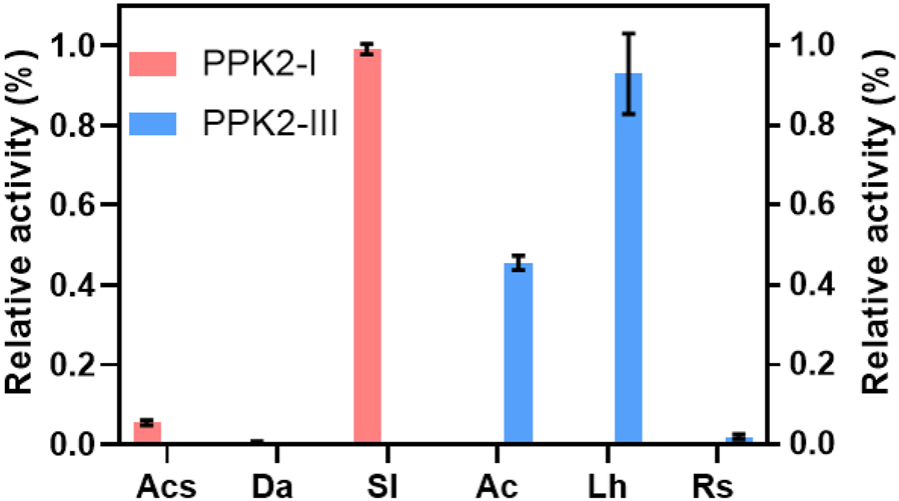
Relative activities of six PPKs. The highest enzyme activity of all PPKs was set as 100% of relative activity. At 37 °C and pH 8.0 (the optimal reaction conditions for AtAdk), the enzymatic activities of PPK2-I to ADP and the enzymatic activities of PPK2-III to AMP were determined. All measurements were carried out in triplicates

To finish the coupling reactions in one pot, it is necessary to ensure that the multiple enzymes could perform at their maximum activity under suitable conditions. The specific activities of six PPKs were assayed under optimal conditions of AtAdk. Three PPKs belonging to Class I used ADP as substrate, while three Class III PPKs used AMP or ADP as substrate. All measured data were shown in Fig. 2 and Table 2. Among three Class I PPKs, SlPPK had the highest relative specific activity compared to the other two PPKs. LhPPK had higher specific activity than AcPPK and RsPPK belonging to PPK2-III. We eventually selected two PPKs with excellent properties that could be efficiently employed in our coupled system.

**Table 2:** Kinetic parameters of the enzyme

	Substrate	Specific activity	*V*_max_	*K*_*m*_	*k*_cat_	*k*_cat_*/K*_*m*_
		(U/mg)	(mM/min)	(mM)	(s^−1^)	
LhPPK	AMP	172.3	0.86	5.70	92	16.1
	ADP	19.1				
AcPPK	AMP	41.5				
RsPPK	AMP	1.7				
SlPPK	ADP	282.9	1.4	0.61	140	229.5
AcsPPK	ADP	4.8				
DaPPK	ADP	0.3				

### Optimization of reaction conditions

The screened LhPPK and SlPPK, which could be employed in the regeneration and production of ATP, were used for coupling experiments according to the above research. To further efficiently obtained products with higher yields, the reaction conditions was studied in the process.

**Fig. 3:**
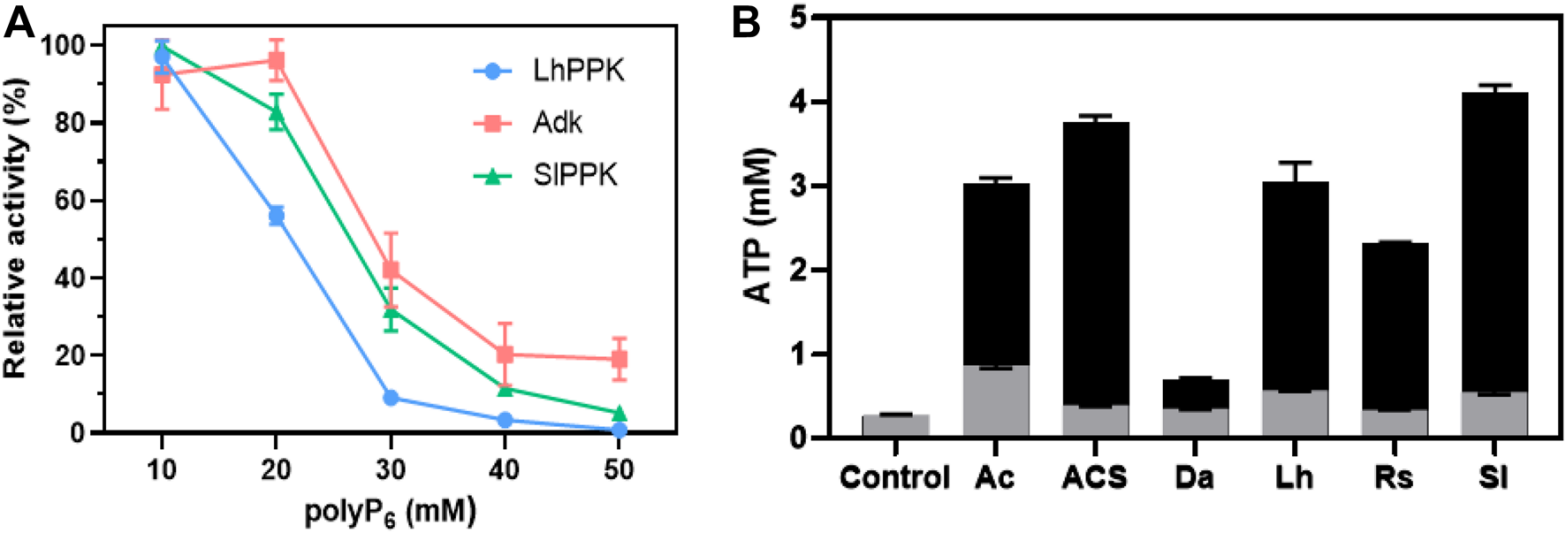
**A** Inhibition of polyphosphate on enzyme activity. The blue, green and red lines represent the effects of polyP_6_ on LhPPK, SlPPK, and AtAdk activity. **B** The utilization of polyP_6_ by different PPKs. The black bar is the amount of ATP produced by the reaction of different PPKs with 1 mM polyP_6_ and 10 mM ADP as substrates. The gray bar is the amount of ATP produced by the reaction of different PPKs with 10 mM ADP as the substrate. All measurements were carried out in triplicates

PolyP is a phosphate donor for the phosphorylation of AMP and ADP. PolyP includes short-chain (3–5 Pi) and long-chain (> 10 Pi) PolyP (Cao et al. 2017). The polyP we used here is polyP_6_. Two points must be considered in the process of using polyP as a phosphate donor. On the one hand, an excessively high concentration of polyP severely inhibits the activity of kinases, such as cytidine kinase (Li et al. 2020a, b), adenosine kinase, polyphosphate kinase, etc. (Fig. 3A). Therefore, the concentration of polyP_6_ in the system must be optimized. On the other hand, different PPKs have different utilization activities regarding polyP chains with different lengths. For example, using polyP_6_ as the phosphate donor, most PPKs can utilize two phosphate groups, whereas some PPKs can utilize three or even four. In the enzymatic reaction process involving polyP, it would be beneficial to the overall reaction catalysis if the PPK could utilize short-chain polyP, as this improves the utilization efficiency of polyP and decreases the cost. Using 1 mM polyP_6_ as the substrate, we finally detected 4 mM ATP after SlPPK- and AcsPPK-catalyzed reactions (Fig. 3B). We accidently discovered that LhPPK had adenylate kinase activity, which was also reported in previous research on PPK properties (Mordhorst et al. 2019). When there was no available phosphate group, LhPPK began to catalyze the reaction to produce ATP and AMP with ADP as the substrate (Fig. 3B and Additional file 1: Figure S3). The properties of adenylate kinase was also existed in other PPKs in this study. Adding excessive amount of polyP to the reaction system not only did not increase the final yield, but also severely inhibited the activity of the enzymes, causing the entire reaction to stagnate. Considering the polyP_6_ utilization of the two PPKs, we intend to add suitable amount of polyP_6_ for ATP regeneration and producion. Thus, the polyP_6_ can be fully exploited, and the inhibition of the reaction can be slowed down to achieve efficient production of ATP.

**Fig. 4:**
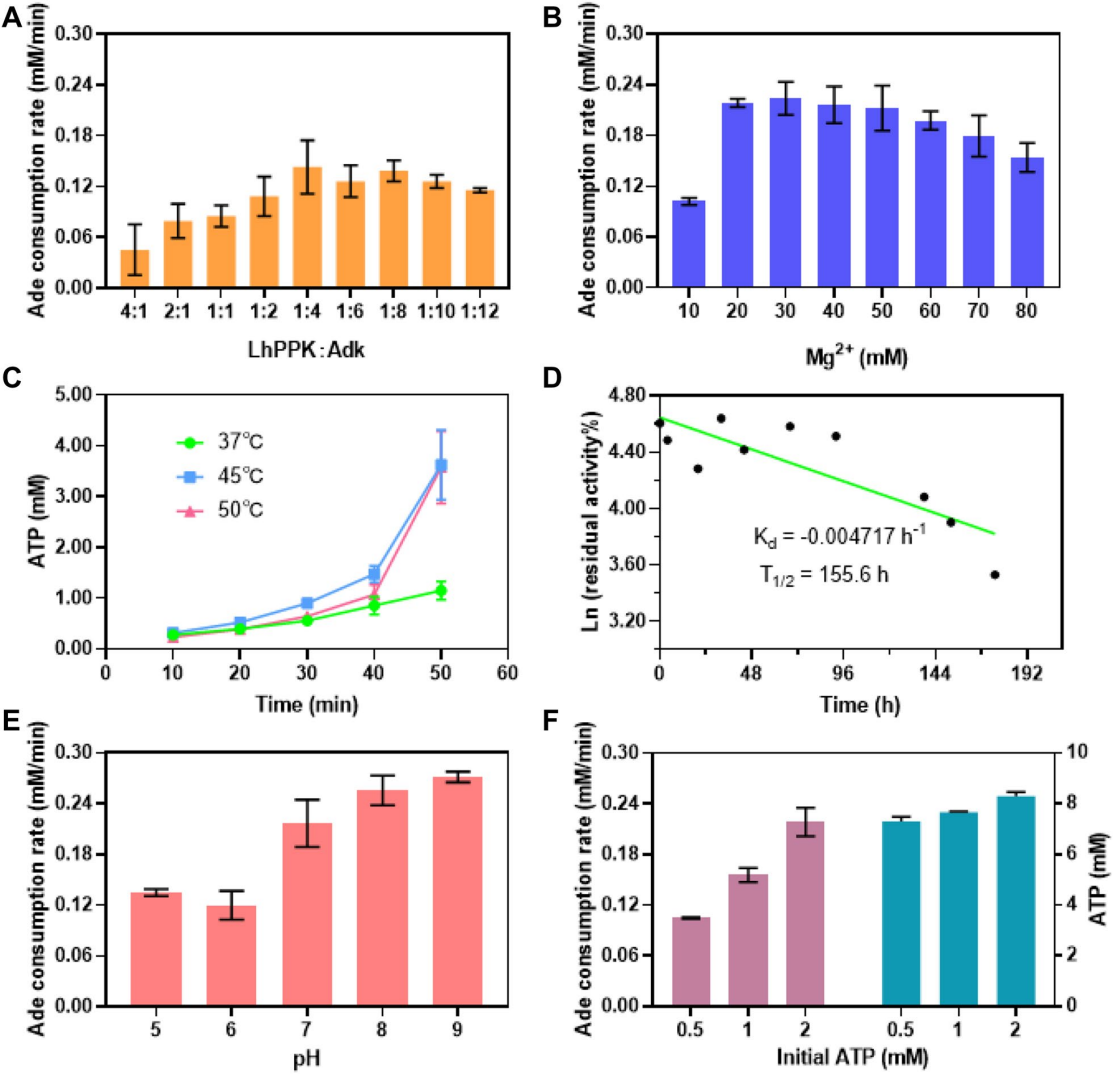
Optimization of first stage conditions. **A** Effect of enzyme ratios on the consumption of adenosine. **B** Effect of Mg^2+^ concentration on the consumption of adenosine. **C** Influence of temperature on the synthesis of ATP. **D** Thermostability of SlPPK at 45 °C. **E** Influence of temperature on the consumption of adenosine. **F** Effect of initial ATP on the consumption of adenosine and final ATP production. All measurements were carried out in triplicates

The two-enzyme coupled reaction in the first stage could be optimized to increase the reaction rate and improve utilization of the substrate. The ratio of the enzyme addition in the first stage is likewise very important for the coupling reaction rate; hence we adjusted the ratio of AtAdk and LhPPK and kept the total amount of enzymes consistent. As shown in Fig. 4A, the rate of adenosine consumption was similar when the enzyme amount ratio of AtAdk:LhPPK was 4:1 and 8:1. However, at a certain amount of total enzyme, more amount of LhPPK could significantly accelerate the accumulation of ADP. We eventually chose 4: 1 of AtAdk: LhPPK to perform the ATP production reaction.

Magnesium ions are very common in kinase-catalyzed reactions as an important cofactor (Adams 2001; Endicott et al. 2012). In this study, in the absence of Mg^2+^, the coupling reaction hardly proceeded. To optimize Mg^2+^ concentration, we, respectively, added 10, 20, 30, 40, and 50 mM Mg^2+^ into the coupling system while maintaining other reaction conditions constant. By calculating the consumption rate of adenosine in 10 min, the optimal Mg^2+^ concentration was determined to be 20 mM (Fig. 4B).

We further optimized the temperature and pH of the reaction system. Although the higher reaction temperature indicates a faster initial catalytic rate, the enzyme could gradually lose its activity during long-term incubation at high temperatures. Therefore, when optimizing the optimal temperature for the reaction, the entire catalytic reaction process must be considered. We chose three different temperatures, namely, 37, 45, and 50 °C for the coupling reaction and finally found that the optimal reaction temperature was 45 °C (Fig. 4C). Moreover, we studied the thermostability of the PPKs. The SlPPK showed half-lifetimes of 155.6 and 68 h at 45 and 50 °C, respectively, which were longer than most of the known PPKs (Fig. 4D, Additional file 1: Figure S4). Furthermore, the SlPPK had a wide pH range (pH 4.0–9.0) (Additional file 1: Figure S5), which indicated substantial application potential. Then, we determined the optimum pH for the first stage reaction by carrying out the experiment at different pH values. pH 8.0 was found to be the optimal pH for the reaction by comparing the adenosine consumption rate (Fig. 4E).

Finally, we optimized the quantity of initial ATP supplement. Evidently, if more ATP was added to start the coupling reaction, the initial consumption rate of adenosine would be faster. However, this had no effect on the final ATP production, regardless of the initial ATP concentration (Fig. 4F). Finally, the coupling reaction was eventually carried out under the conditions of enzyme ratio of AtAdk: LhPPK = 4: 1, magnesium ion concentration of 20 mM, temperature of 45 °C, and pH of 8.0.

### One-pot synthesis of ATP

**Fig. 5:**
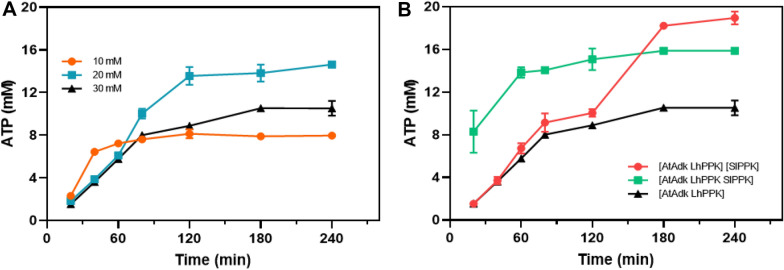
Production of ATP under optimized conditions. **A** Production of ATP at different initial adenosine concentrations when only AtAdk and LhPPK were added to the reaction system. The orange, blue and black lines represent the reaction system of 10, 20 and 30 mM adenosine. **B** Production of ATP at different conditions with 30 mM adenosine as substrate. Black line: Only two enzymes of AtAdk and LhPPK were added to these system. Green line: Three enzymes of AtAdk, LhPPK and SlPPK were added simultaneously to the system. Red line: Two enzymes of AtAdk and LhPPK were added to the system at the beginning, and the SlPPK was added at 120 min. All measurements were carried out in triplicates

After optimization of the coupling reaction system, ATP production was performed, and the content changes of AMP, ADP, and ATP were measured during the process. Based on the above research, we determined the substrate of 20 mM polyP_6_ and 0.5 mM ATP remaining unchanged, and further studied the production of ATP under different adenosine concentrations (Fig. 5). LhPPK had a low activity on ADP. ADP began to accumulate quickly, but ATP was slowly generated when the adenosine was completely consumed. When adenosine concentration was low, such as 10 and 20 mM, the reaction equilibrium could be slowly reached without the addition of SlPPK. The ATP content reached the maximum of 8.1 and 14.6 mM. The yields were 76.0 and 70.5%, respectively. Because the supply of phosphate groups was sufficient, adding three enzymes at the same time would speed up the reaction rate and quickly reached the equilibrium of the reaction, and the final concentration of ATP generated was consistent. As the substrate concentration continued to increase to 30 mM, the AMP and ADP in the system cannot be converted into ATP anymore, because there was no short-chain polyP available for LhPPK. The ATP production yield was only 33.5%. When the three enzymes were added simultaneously at the beginning, the yield of ATP production was 51.2%. Therefore, a strategy of adding two PPKs sequentially was designed to improve the utilization of polyP_6_. The adenosine was completely consumed in 2 h and the SlPPK was then added into the system. AMP and ADP cannot be converted completely by PPK due to the thermodynamic equilibrium as well as substrate and product inhibition. The ATP content reached 18.9 mM at 4 h, and the yield was 61.3%. The high performance liquid chromatogram of the content changes of ATP, ADP and AMP in the production system with 30 mM adenosine as a substrate are shown in Fig. S6.

According to the conservation of the number of phosphate groups transferred, the complete conversion of 1 mM adenosine into 1 mM ATP required 3 mM phosphate groups. If a PPK could utilize three phosphate groups of polyP_6_, 10, 20, and 30 mM adenosine reactions required 10, 20, and 30 mM polyP_6_, respectively. If a PPK could utilize 4 phosphate groups in polyP_6_, 10, 20, and 30 mM adenosine reactions required 7.5, 15, and 22.5 mM polyP_6_, respectively. Therefore, for 10 and 20 mM adenosine, the supply of phosphate groups was sufficient. For 30 mM adenosine, when the three enzymes were added at the same time, SlPPK and LhPPK would utilize the phosphate group from polyP_6_ at the same time, followed by polyP_5_, polyP_4_ and so on. It was likely to cause the system quickly using up the polyP_4_ that could be used by LhPPK, and the AMP and even adenosine had not reacted completely, thereby reducing the utilization efficiency of polyP_6_.

Using different concentration of adenosine, we found that as the concentration increased, the conversion rate to ATP gradually decreased. Adenosine had almost no effect on the activity of adenosine kinase, while polyP_6_, sodium trimetaphosphate (polyP_3_), and even pyrophosphate (PPi) had a significant inhibitory effect on the activities of AtAdk, LhPPK, and SlPPK. Therefore, the concentration of polyP6 being added to the reaction mixture must be optimal so that it does not significantly affect the enzyme activity. If the substrate concentration is further increased, different feeding strategies can be applied to ensure polyP supply. Furthermore, the generated ATP seemed to have an effect on the progress of the reaction. The reason might be that ATP inhibits PPK products at high concentrations, or the equilibrium problem occurs. Nevertheless, the ATP synthesis system constructed in vitro provides a novel approach for the production of ATP.

## Conclusions

In this study, we successfully constructed a system that catalyzes the production of ATP by coupling multiple enzymes. The presence of polyP severely inhibits the progress of the reaction, while the generated ATP likewise affects the enzyme activity and reaction equilibrium. Fortunately, SlPPK can utilize four phosphate groups of polyP_6_, which significantly reduces the amount of polyP_6_ added. Many synthetic reactions coupled with ATP regeneration require a suitable PPK to be found, based on the acid or alkalinity of the reaction. The specific activity of SlPPK in this article is relatively high, and it operates at pH 4.0–9.0. SlPPK, which exhibits excellent activity over a wide pH range, might be a universal PPK for in vitro synthesis systems that require ATP regeneration. And then, solving the problem of PPK enzyme activity being inhibited might be of great help to the in vitro synthesis of ATP.

### Supplementary Information

**Additional file 1**. Codon-optimized DNA sequences of LhPPK, SlPPK, AcsPPK, DaPPK, RsPPK, AcPPK, and Adk. **Figure S1.** SDS-PAGE analysis of recombinant enzymes. Figure S2: (A) Time course of adenosine analyzed by HPLC. **Figure S2.** (A) Time course of adenosine analyzed by HPLC. **Figure S3.** Adenylate kinase properties of LhPPK. **Figure S4.** Thermostability of SlPPK at 50 °C. **Figure S5.** Relative enzyme activity of SlPPK at different pH values.

## Data Availability

All data generated or analyzed during this study are included in this article and the supplementary information file.

## Abbreviations

ATP: Adenosine triphosphate
ADP: Adenosine diphosphate
AMP: Adenosine monophosphate
polyP_6_: Hexametaphosphate
PPi: Pyrophosphate
PPK: Polyphosphate kinase
Adk: Adenosine kinase
PB buffer: Phosphate buffer
Tris–HCl buffer: Tris(hydroxymethyl)aminomethane buffer
LB medium: Luria–Bertani medium
SDS-PAGE: Sodium dodecyl sulfate polyacrylamide gel electrophoresis
PCR: Polymerase chain reaction
IPTG: Isopropyl β-d-1-thiogalactopyranoside
HPLC: High-performance liquid chromatography
