# Supplementary material for: In vitro biosynthesis of ATP from adenosine and polyphosphate
Source: Bioresour Bioprocess. 2021 Nov 29;8(1):117. doi: 10.1186/s40643-021-00469-0 (PMC10992290; doi:10.1186/s40643-021-00469-0)

**Supporting Information Description**

**In vitro Biosynthesis of ATP from Adenosine and Polyphosphate**

Chuanqi Sun^a^, Zonglin Li*^, a^, Xiao Ning^a^, Wentian Xu^a^, Yi-Heng Percival Zhang^b^, Zhimin Li*^, a, c, b^

a. State Key Laboratory of Bioreactor Engineering, East China University of Science and Technology, 130 Meilong Road, Shanghai 200237, China

b. Tianjin Institute of Industrial Biotechnology, Chinese Academy of Sciences, 32 West 7th Avenue, Tianjin Airport Economic Area, Tianjin 300308, China

c. Shanghai Collaborative Innovation Center for Biomanufacturing Technology, 130 Meilong Road, Shanghai 200237, China

* **Corresponding author:**

Zhimin Li

Telephone: +86-21-64252095

**E-Mail:** [lizm@ecust.edu.cn](mailto:lizm@ecust.edu.cn)

**Supplementary Materials and methods**

**Codon-optimized DNA sequences of LhPPK**

ATGACGCGCAAAGAAAGCCCGCCGATGGAAGCCTTCAAGAAGTACCGCATCAGCCCGAAGAACACGCTGAAAGACATCGATCCGAGCGACAAGCCGTTCAGCTTCGGCACGAAAAAGGATGAGCTGCAGCGTCTGGATGAACTGGCGGTTGAACTGGACGATCTGCAGAATACGCTGTACGCGGGCAAACGCCGCAAAGTTCTGCTGATTCTGCAAGGTCTGGACACCAGCGGCAAAGACGGTACCATCCGCTGGGTGTTTAGTCGTACCAGCCCGCTGGGTGTGCATGTTGCGGCCTTCAAAGCCCCGAGCGAACGCGAACGCGCCCATGATTTCCTCTGGCGCTGCCACGCGGTTGTTCCAGCGAACGGCGAACTGACCGTGTGGAATCGCAGCCATTACGAAGACGTTCTGGTGCCGCCAGTGGAGGGTTGGATCGATAAAGCGGAAACCCAGCGCCGCTATGGCCATATCAACGATTTCGAGCGTCTGCTGAGCGAAACGGGTACCACCATCGTGAAGTGCATGCTGCACATCAGCAACGAAGAGCAGCGCGAACGTCTGCAAGACCGTCTGAAGGATCCGGGCAAGAACTGGAAATTCGCGGCGGATGATCTGAGCACCCGCGCCAAATGGAACGACTACCAGCGTGCCTACGAACAAGCGCTGCAAGCGACCAGTACCCCACATGCGCCGTGGTACGTTATCCCAGCCAACAGCAAACGCCATCGCAATCTGATGGTTGCGCAGCTGCTGGTTCAGACGCTGCGCGACATGAAACTGCGTCTGCCACCGCCGAATCTGGCCTTTAAGGATCTGGTGGTGAACTAA

**Codon-optimized DNA sequences of SlPPK**

ATGAAGAAAAACATCTACAAAAAAGAACTGTACAAACTGCAGGTTGAACTGGTTAAATTCCAGAAATACGTTATCGAAGAAAACGTTGCGGTTTGCCTGGTTCTGGAAGGCCGTGATACCGCGGGTAAAGATGGCACCATCAAACGTTTCACCGAACACCTGAGCCCGCGTGAAGCGCGTACCGTTGCGCTGGGCGTTCCGAGCGATAAAGAAAAGAAATCTTGGTACTTCCAGCGTTACGTTCCGCACCTGCCGTCTGCGGGCGAAATCGTTTTCTTCAACCGTAGCTGGTACAACCGCGCGGGCGTTGAAAAAGTTATGGGCTTCTGCACCAAAAAACAGTACAAAGCGTTCATGGAAGAAGTTGGTTCCTTCGAACAGATGCTGACCCACAGCAACATCCGTTTCTTCAAATACTACCTGGATATCACCAAAAAAGAACAGAAAAAACGCCTGGAAGCGCGTAAAACCGATCCGCTGAAACAGTGGAAACTGTCCCCGATCGATGCGAAAGCGCAGAAAATGTGGGATGCGTACAGCAAAGCGCGTGATGATATGTTCAACAAAACCAGCTTCATCTACGCGCCGTGGTACGTTGTTCACACCGATGATAAAAAAGAAGCGCGTATCAACATCATGAAACACTTCCTGAGCCTGAACGACTACCCGGATAAAGATAAAGCGCTGCTGGTTTACGATCACGATGTTATCTGCAAATTCGACCCGGTTTGCTACGAAAAAGAAATGATCGCGCCGTAA

**Codon-optimized DNA sequences of AcsPPK**

ATGAACCACAGCGTTGATAAAGCGCAGAACCAGTACAAAGCGACCCTGCACCTGCTGCAGATCGAACTGGTTAAACTGCAGCGTCACATCATCAAACGTGGTGAACGTATCCTGCTGATCCTGGAAGGCCGTGATGGCGCAGGTAAAGATAGCACCATCAAACGTATCATCGCGCACCTGAGCCCGCGTGAAACCCGTGTTGTTGCGCTGCCGAAACCGTCTGATCGTGAACAGACCGAATGGTACTTCCAGCGTTACGTTGCGCACCTGCCGGCGGCGGCGGAATTCGTTATCTTCAACCGTAGCTGGTACAACCGTGCGGGTGTTGAAAAAGTTATGGGCTTCTGCTCTGATCGTGATTACGAAGAATTCTTCGCGGAAGTTAACGATTTCGAAAGCATGCTGACCCGTAGCGGCATCCTGCTGCGTAAATACTATCTGGATATCACCAAAAAAGAACAGAAAGCGCGTCTGGCGGCGCGTCGTGATGATCCGCTGAAACAGTGGAAAATCAGCCCGATCGATGAACAGGCGCTGAAACACTGGAAAGATTACTCTGAAGCTCGTAACGTTATGTTCGCGCGTTCTCACACCCACCTGTGCCCGTGGACCATCGTTCACAGCGATGAAAAGAAAACCGCACGTATCCAGCTGATCAAAGATTTCCTGTCTCGTCTGGAATACGCGGATAAAGATATCGATCTGCTGGCGCCGGATCGTGAAGTTGTTTTCGATTACGATCCGGCGTACCTGAAAAACGATATGATCGCGCCGTAA

**Codon-optimized DNA sequences of DaPPK**

ATGAGCAAAGAAAAAGAAAACGAAAAACAGGAACTGTGCAAACTGTGGATCGAACTGGTTAAATTCCAGAACACCCTGATCAAAAAAGAAGAAAAAGTTCTGCTGATCCTGGAAGGCCGTGATAGCGCGGGTAAAGACGGCATGATCCGACCATCACCCGCCACCTGAGCCCGCGTGAAACCCGCGTGTTCGCGGTGAGCAAACCGACCGACAAAGAAGCGAAAGAATGGTACTTCCAGCGTTTCGTTCCGCACCTGCCGGCGCCGTGCGAATTCGTGCTGTTCAACCGCAGCTGGTACAACCGTGCGGGTGTTGAACGTGTGATGGGCTTCTGCACCAAAAAAGAATACGAACAGTTCTACGAAGATGTTATCTACTTCGAAAACCTGCTGCTGAACGCGGGTATCAAAATGTTCAAATTCTATCTGGATATCGATAAAAAAGAACAGGAAAAACGTCTGGAATCCCGTAAAAAAGATCCGCTGAAACAGTGGAAAGAAAGCCCGGTTGATGATGCAGCGATCAAACACTTCGATGATTACACCCAGTCCCGTAACGAAATGTTCGAACGTACCCACACCCCGCAGAGCCCGTGGGTTATCGTTAACGCGAACGACAAACACAAAGCGCGTCTGAACCTGATCAAATACTTCCTGCTGAACGTGGACTACAAAGAAAAGAACGAAAAAATCCTGAACGTGGACCCGAACATCGTTGTTGTTTACAACAAAAACTTCTTCATCAAATAA

**Codon-optimized DNA sequences of RsPPK**

ATGGCGACGAAAGCCCCAGATGGTGCCTACTTTCAGTACGCCGATGCGGGCAGCAAAAAACGCGCCTTCGATCTGAGTGCCATCGATCCGGGCGCCAAACCGTTCAGCACCGGCGATAAAGTGGCCGATAAAGCCGCCGTTGATGCGCTGGCGCTGGAACTGGATGAGCTGCAGAATCTGTTCTACGCCGATCGCCGCTTTAAACTGCTGGTGGTTCTGCAAGGCACCGATACCAGCGGCAAAGATGGTACGCTGCGCGGCGTTTTCGGCCAGATGAGCGCGCTGGGCGTTCAGACCGTTGGTTGGAAAGCGCCAACGGCCCCAGAACGCGACCATGACTATCTGTGGCGCATCCACCAGAAAGTGCCGGGCGCGGGCGAAGTGATGGTTTTCAACCGCAGCCACTACGAGGATGTGCTGGTTCCGGTGGTTAATGGCTGGATCACCCCACAGCAGACCCGCCAGCGTTATGCGCAGATCAACGATTTCGAGCGCATGCTGACCGAAACCGGCACCGTGGTTCTGAAGTTCATGCTGCACATCAGCAAGGACGAGCAGCGCATCCGTCTGCAAGAACGCCTCGATGATCCGACCAAGAACTGGAAGTTCGCCGCCGATGATCTGACCGTGCGCAAACAGTGGAAGGCCTACCAGCAAGCCTATGCCGATGCCATTGCCGCGACGGGTACCCGTTGGGCGCCATGGACGGTTGTTCCGGCCGATAGCAAAACCCACCGCAACCTCATGATCGCCACGCTGGTGAAACGTGCGCTGGTGGGTCTGAAGCTGCGCTACCCACCAGCCGGCGAAGATCTGACCAAACTCCGCGTGCGCTAA

**Codon-optimized DNA sequences of AcPPK**

ATGAGCAGCCCGTTCGATAACCGCTTCGATAGCCCGGACAAGAGTCTGGCCAAACTGTGGCGCGATTGGCAACCGCAAGCCGCCAAAGATCGTAAAGCGCCAAGCCTCGCCAAATTCGACCCGAGTGCCAAACCGTTCAGCCAAGGCGGTAAGGCGGAGGATAAAGCCGCGGTTGAAGCGCTGGCGGTTGAGCTGGACACGCTGCAGAATCTGTTCTACGCCGACAAGCGCTACAAACTGCTGGTGGTTCTGCAAGGCACCGATACCAGCGGTAAAGATGGCACCATCCGTGGCGTTTTTGGCCGCATGAGCGCGCTGGGTGTTCATGCCGTTGGCTGGAAAGCGCCGACGGAAACCGAACGCGCCCATGATTATCTGTGGCGCATCCACCAGCAAGTTCCGCAAGATGGCGACATCACCGTGTTCAACCGCAGCCATTACGAAGACGTTCTGGTGCCGGTGGTTAACGGTTGGATCACCCCGGCGCAGCAACAACAGCGTCTGGCCCATATCAACGACTTCGAGCGCATGCTGAGCGAAACCGGCACCATCGTTCTGAAGTTTCTGCTGCACATCAGCCCGGATGAACAGCGTCTGCGTCTGCAAGAACGTCTGGATGATCCGGCCAAACACTGGAAGTTCAGCATGGGCGACATCGAAGTGCGCAAACAGTGGGACGATTACCGTCGCGCGTACGATACGCTGCTGCATGCCACCCATACCCCATGGGCCCCATGGACGATCGTTCCGGCCAACAGCAAGACGCACCGCAATCTGATGATCGCGACGGTTCTGCGCCAAGTTCTGCAAAATCTGAATCTGCGCTATCCACCGGGCGATCCGCTGCTGGTTAACTTCAAGGTGGAATAA

**Codon-optimized DNA sequences of Adk**

ATGGCGAGCTCTTCTAACTACGATGGCATCCTGCTGGGTATGGGTAACCCACTGCTGGACATCTCTGCAGTTGTTGACGATGAATTTCTGACCAAGTACGATATCAAACTGAACAACGCAATCCTGGCAGAAGATAAACACCTGCCGATGTATGACGAAATGTCTTCTAAATTCAACGTTGAATATATCGCTGGCGGTGCCACCCAAAACTCCATCAAAGTTGCACAGTGGATGCTGCAGATCCCTGGCGCAACCTCTTACATGGGTAGCATCGGCAAAGACAAATATGGCGAGGCAATGAAAAAAGACGCAACCGCTGCGGGTGTTAATGTGCACTACTACGAAGACGAGAGCGCGCCGACTGGTACCTGTGGTGTCTGTGTGGTTGGTGGCGAACGTTCTCTGATCGCGAACCTGAGCGCAGCTAACTGCTATAAAGTTGATCACCTGAAAAAACCGGAAAACTGGGCGCTGGTGGAAAAAGCTAAATTCTACTACATTGCGGGCTTTTTCCTGACGGTTAGCCCGGAATCTATCCAGCTGGTTTCCGAACACGCGGCTGCTAACAACAAAGTATTCACTATGAACCTGTCCGCGCCGTTCATCTGCGAATTCTTCAAGGACGTTCAGGAAAAGTTTCTGCCGTACATGGACTTCGTGTTCGGTAATGAGACCGAAGCACGTACCTTCTCTCGCGTGCACGGTTGGGAAACTGAAGATGTTGAACAGATCGCTATTAAAATCTCCCAGCTGCCAAAAGCTACTGGCACTTATAAACGTACCACCGTGATTACTCAGGGTGCTGATCCGGTCGTCGTAGCAGAGGACGGTAAAGTGAAAAAGTATCCGGTTATCCCGCTGCCGAAAGAGAAACTGGTGGATACCAACGGTGCAGGTGACGCTTTTGTTGGTGGTTTTATGAGCCAGCTGGTTAAAGAAAAATCTATCGAAGAATGCGTCAAAGCTGGCTGCTATGCGAGCAACGTCGTCATTCAGCGCTCTGGCTGCACCTATCCGGAGAAACCGGACTTCAAC

Supplementary Figure captions:

**Figure S1:** SDS-PAGE analysis of recombinant enzymes. M: protein marker; Sl: SlPPK; Acs: AcsPPK; Da: DaPPK; Lh: LhPPK; Rs: RsPPK; Ac: AcPPK.

**Figure S2:** (A) Time course of adenosine analyzed by HPLC. Adenosine exhibits a retention time of approximately 14.4 min. (B) Time course of ATP, ADP, and AMP analyzed by HPLC. Their retention times were 4.8, 5.9, and 11.6 min, respectively.

**Figure S3:** Adenylate kinase properties of LhPPK. The blue line represents the changes in the content of AMP, ADP, and ATP after 10 minutes.

**Figure S4:** Thermostability of SlPPK at 50 °C.

**Figure S5:** Relative enzyme activity of SlPPK at different pH values.

**Figuer S6:** High performance liquid chromatogram of the changes in the content of ATP, ADP and AMP in the production system with 30mM adenosine as substrate.

**Figure S1:**


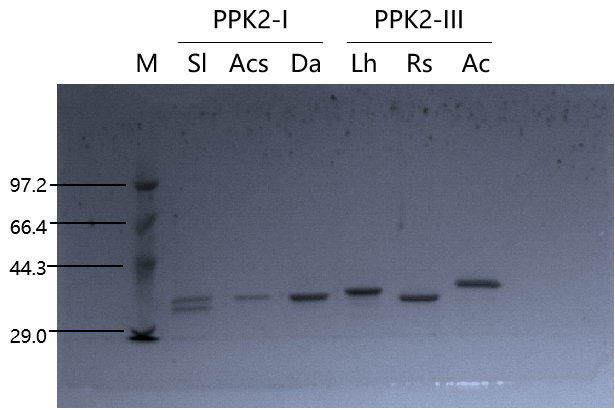


**Figure S2:**
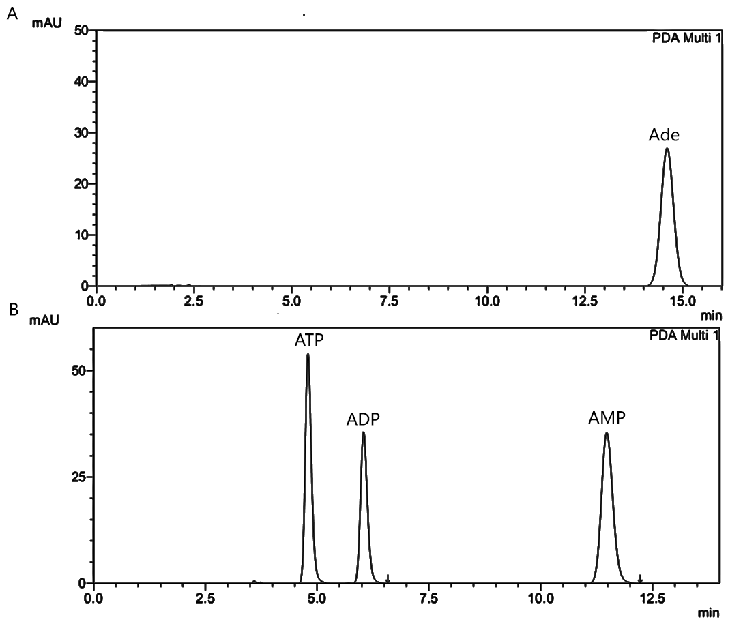


**Figure S3:**


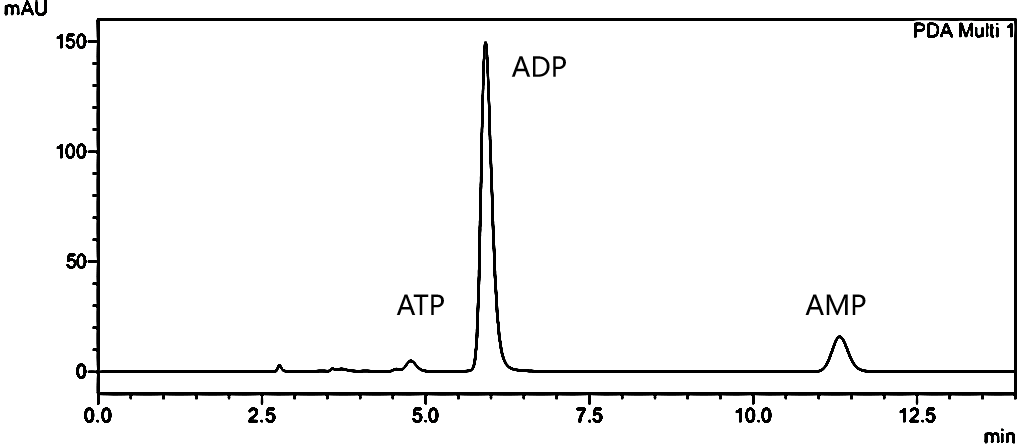


**Figure S4:**


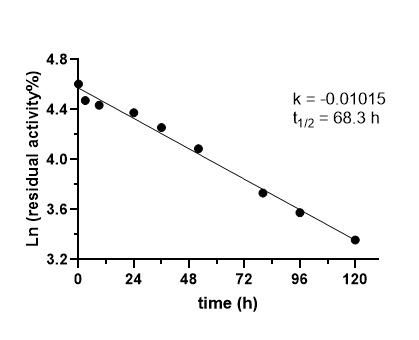


**Figure S5:**


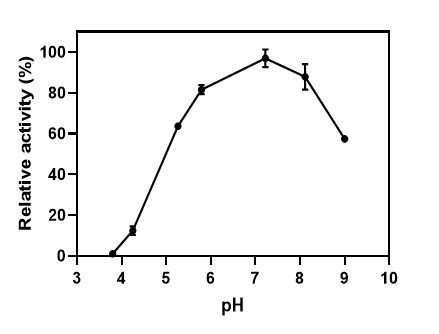


**Figure S6:**


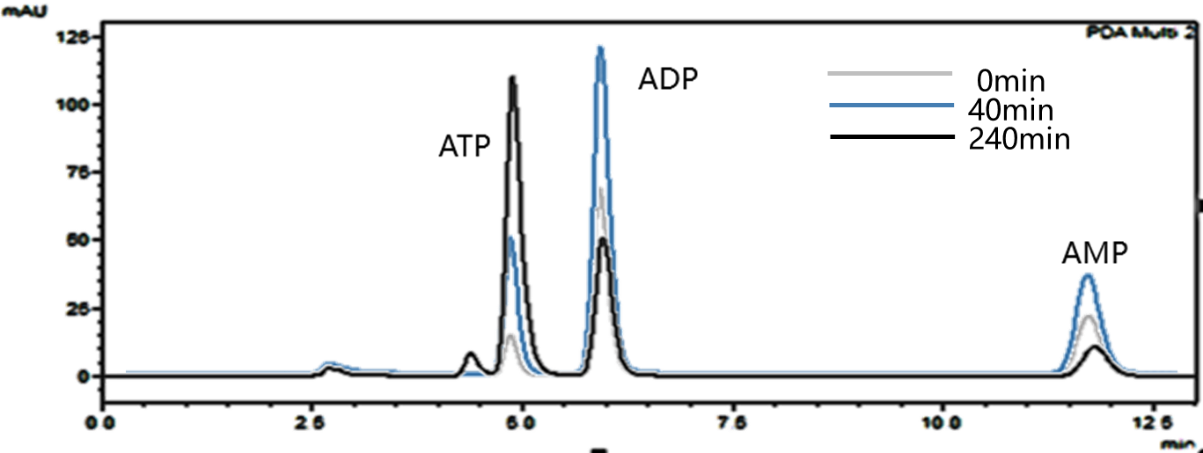

Supplement: Supplementary file 1 — Additional file 1. Codon-optimized DNA sequences of LhPPK, SlPPK, AcsPPK, DaPPK, RsPPK, AcPPK, and Adk. Figure S1. SDS-PAGE analysis of recombinant enzymes. Figure S2: (A) Time course of adenosine analyzed by HPLC. Figure S2. (A) Time course of adenosine analyzed by HPLC. Figure S3. Adenylate kinase properties of LhPPK. Figure S4. Thermostability of SlPPK at 50 °C. Figure S5. Relative enzyme activity of SlPPK at different pH values. [file 40643_2021_469_MOESM1_ESM.docx]
